# Interim Effectiveness of Updated 2023–2024 (Monovalent XBB.1.5) COVID-19 Vaccines Against COVID-19–Associated Emergency Department and Urgent Care Encounters and Hospitalization Among Immunocompetent Adults Aged ≥18 Years — VISION and IVY Networks, September 2023–January 2024

**DOI:** 10.15585/mmwr.mm7308a5

**Published:** 2024-02-29

**Authors:** Jennifer DeCuir, Amanda B. Payne, Wesley H. Self, Elizabeth A.K. Rowley, Kristin Dascomb, Malini B. DeSilva, Stephanie A. Irving, Shaun J. Grannis, Toan C. Ong, Nicola P. Klein, Zachary A. Weber, Sarah E. Reese, Sarah W. Ball, Michelle A. Barron, Allison L. Naleway, Brian E. Dixon, Inih Essien, Daniel Bride, Karthik Natarajan, Bruce Fireman, Ami B. Shah, Erica Okwuazi, Ryan Wiegand, Yuwei Zhu, Adam S. Lauring, Emily T. Martin, Manjusha Gaglani, Ithan D. Peltan, Samuel M. Brown, Adit A. Ginde, Nicholas M. Mohr, Kevin W. Gibbs, David N. Hager, Matthew Prekker, Amira Mohamed, Vasisht Srinivasan, Jay S. Steingrub, Akram Khan, Laurence W. Busse, Abhijit Duggal, Jennifer G. Wilson, Steven Y. Chang, Christopher Mallow, Jennie H. Kwon, Matthew C. Exline, Cristie Columbus, Ivana A. Vaughn, Basmah Safdar, Jarrod M. Mosier, Estelle S. Harris, Jonathan D. Casey, James D. Chappell, Carlos G. Grijalva, Sydney A. Swan, Cassandra Johnson, Nathaniel M. Lewis, Sascha Ellington, Katherine Adams, Mark W. Tenforde, Clinton R. Paden, Fatimah S. Dawood, Katherine E. Fleming-Dutra, Diya Surie, Ruth Link-Gelles, CDC COVID-19 Vaccine Effectiveness Collaborators, CDC COVID-19 Vaccine Effectiveness Collaborators, Shekhar Ghamande, Robert Gottlieb, Tresa McNeal, Catherine Raver, William Bender, Linda Fletcher, Phillip Heaton, Sheryl Kane, Charlene McEvoy, Sunita Thapa, Gabriela Vazquez-Benitez, Anne Frosch, Lois E Lamerato, Mayur Ramesh, Julie Arnofer, Harith Ali, Johns Hopkins, Bradley Crane, Padma Dandamudi, Kristin Goddard, John Hansen, Julius Timbol, Ousseny Zerbo, Katie Allen, Thomas Duszynski, William Fadel, Colin Rogerson, Nida Qadir, Catia Chavez, Bryant Doyle, David Mayer, Suchitra Rao, Carolina Rivas, Nicholas J. Johnson, Adrienne Baughman, Cara T. Lwin, Jillian P. Rhoads, Kelsey N. Womack, Margaret Dunne, Allison Ciesla, Josephine Mak, Morgan Najdowski, Caitlin Ray

**Affiliations:** ^1^Coronavirus and Other Respiratory Viruses Division, National Center for Immunization and Respiratory Diseases, CDC; ^2^Vanderbilt University Medical Center, Nashville, Tennessee; ^3^Westat, Rockville, Maryland; ^4^Division of Infectious Diseases and Clinical Epidemiology, Intermountain Healthcare, Salt Lake City, Utah; ^5^HealthPartners Institute, Minneapolis, Minnesota; ^6^Kaiser Permanente Center for Health Research, Portland, Oregon; ^7^Indiana University School of Medicine, Indianapolis, Indiana; ^8^Regenstrief Institute Center for Biomedical Informatics, Indianapolis, Indiana; ^9^University of Colorado School of Medicine, Aurora, Colorado; ^10^Kaiser Permanente Vaccine Study Center, Kaiser Permanente Northern California Division of Research, Oakland, California; ^11^Department of Biomedical Informatics, Columbia University Irving Medical Center, New York, New York; ^12^New York-Presbyterian Hospital, New York, New York; ^13^General Dynamics Information Technology, Falls Church, Virginia; ^14^University of Michigan, Ann Arbor, Michigan; ^15^Baylor Scott & White Health, Texas; ^16^Baylor College of Medicine, Temple, Texas; ^17^Intermountain Medical Center, Murray, Utah; ^18^University of Utah, Salt Lake City, Utah; ^19^University of Iowa, Iowa City, Iowa; ^20^Wake Forest School of Medicine, Winston-Salem, North Carolina; ^21^Johns Hopkins University School of Medicine, Baltimore, Maryland; ^22^Hennepin County Medical Center, Minneapolis, Minnesota; ^23^Montefiore Medical Center, Albert Einstein College of Medicine, New York, New York; ^24^University of Washington, Seattle, Washington; ^25^Baystate Medical Center, Springfield, Massachusetts; ^26^Oregon Health & Science University, Portland, Oregon; ^27^Emory University, Atlanta, Georgia; ^28^Cleveland Clinic, Cleveland, Ohio; ^29^Stanford University School of Medicine, Stanford, California; ^30^Ronald Reagan UCLA Medical Center, Los Angeles, California; ^31^University of Miami, Miami, Florida; ^32^Washington University in St. Louis, St. Louis, Missouri; ^33^The Ohio State University, Columbus, Ohio; ^34^Texas A&M University College of Medicine, Dallas, Texas; ^35^Henry Ford Health, Detroit, Michigan; ^36^Yale University School of Medicine, New Haven, Connecticut; ^37^University of Arizona, Tucson, Arizona; ^38^Influenza Division, National Center for Immunization and Respiratory Diseases, CDC.; Baylor Scott & White Health; Baylor Scott & White Health; Baylor Scott & White Health; Baylor Scott & White Health; Emory University; HealthPartners; HealthPartners; HealthPartners; HealthPartners; HealthPartners; HealthPartners; Hennepin County Medical Center; Henry Ford Health; Henry Ford Health; Intermountain Healthcare; Kaiser Permanente Center for Health Research; Kaiser Permanente Center for Health Research; Kaiser Permanente Northern California; Kaiser Permanente Northern California; Kaiser Permanente Northern California; Kaiser Permanente Northern California; Regenstrief Institute; Regenstrief Institute; Regenstrief Institute; Regenstrief Institute; University of California, Los Angeles; University of Colorado; University of Colorado; University of Colorado; University of Colorado; University of Miami; University of Washington; Vanderbilt University Medical Center; Vanderbilt University Medical Center; Vanderbilt University Medical Center; Vanderbilt University Medical Center; Westat; CDC; CDC; CDC; CDC.

**Summary**

**What is already known about this topic?**

In September 2023, CDC’s Advisory Committee on Immunization Practices recommended updated 2023–2024 (monovalent XBB.1.5) COVID-19 vaccination for all persons aged ≥6 months to prevent COVID-19, including severe disease. Few estimates of updated 2023–2024 vaccine effectiveness against medically attended COVID-19 are available.

**What is added by this report?**

Receipt of an updated COVID-19 vaccine dose provided increased protection against COVID-19–associated emergency department and urgent care encounters and hospitalization compared with no receipt of an updated vaccine dose among immunocompetent U.S. adults during a period of multiple cocirculating SARS-CoV-2 Omicron lineages.

**What are the implications for public health practice?**

These findings support CDC recommendations for updated 2023–2024 COVID-19 vaccination. All persons aged ≥6 months should receive updated 2023–2024 COVID-19 vaccine.

## Abstract

In September 2023, CDC’s Advisory Committee on Immunization Practices recommended updated 2023–2024 (monovalent XBB.1.5) COVID-19 vaccination for all persons aged ≥6 months to prevent COVID-19, including severe disease. However, few estimates of updated vaccine effectiveness (VE) against medically attended illness are available. This analysis evaluated VE of an updated COVID-19 vaccine dose against COVID-19–associated emergency department (ED) or urgent care (UC) encounters and hospitalization among immunocompetent adults aged ≥18 years during September 2023–January 2024 using a test-negative, case-control design with data from two CDC VE networks. VE against COVID-19–associated ED/UC encounters was 51% (95% CI = 47%–54%) during the first 7–59 days after an updated dose and 39% (95% CI = 33%–45%) during the 60–119 days after an updated dose. VE estimates against COVID-19–associated hospitalization from two CDC VE networks were 52% (95% CI = 47%–57%) and 43% (95% CI = 27%–56%), with a median interval from updated dose of 42 and 47 days, respectively. Updated COVID-19 vaccine provided increased protection against COVID-19–associated ED/UC encounters and hospitalization among immunocompetent adults. These results support CDC recommendations for updated 2023–2024 COVID-19 vaccination. All persons aged ≥6 months should receive updated 2023–2024 COVID-19 vaccine.

## Introduction

On September 12, 2023, CDC’s Advisory Committee on Immunization Practices recommended updated 2023–2024 COVID-19 vaccination with a monovalent XBB.1.5–derived vaccine for all persons aged ≥6 months to prevent COVID-19, including severe disease (*1*). Although 1 updated vaccine dose is recommended for most persons aged ≥5 years, vaccination coverage with updated vaccines has remained low,* including among those at highest risk for severe disease, such as adults aged ≥65 years. Thousands of persons in the United States continue to be hospitalized with COVID-19 each week, including approximately 31,000 during January 7–13, 2024, despite endemicity and increased population immunity to SARS-CoV-2.^†^ This analysis estimated updated COVID-19 vaccine effectiveness (VE) during September 2023–January 2024^§^ among immunocompetent adults aged ≥18 years against COVID-19–associated emergency department (ED) or urgent care (UC) encounters in one CDC VE network and VE against COVID-19–associated hospitalization in two CDC VE networks.

## Methods

### Data Collection

Methods for Virtual SARS-CoV-2, Influenza, and Other respiratory viruses Network (VISION) and Investigating Respiratory Viruses in the Acutely Ill (IVY) VE analyses have been described (*2*,*3*). VISION is a multisite, electronic health records (EHR)–based network including 369 EDs and UCs and 229 hospitals in eight states^¶^ that uses a test-negative, case-control design to estimate COVID-19 VE. Eligible patients must receive molecular testing (e.g., real-time reverse transcription–polymerase chain reaction [RT-PCR] testing) for SARS-CoV-2 during the 10 days preceding or up to 72 hours after a COVID-19–associated ED/UC encounter or hospital admission.** COVID-19 vaccination history is ascertained from state or jurisdictional registries, EHRs, and, in a subset of sites, medical claims data.

IVY is a multisite, inpatient network including 26 hospitals in 20 U.S. states^††^ that uses a test-negative, case-control design to prospectively enroll patients with COVID-19–like illness (CLI)^§§^ who receive testing for SARS-CoV-2 within 10 days of illness onset and 3 days of hospital admission. Nasal swabs are collected for central RT-PCR testing for SARS-CoV-2 at Vanderbilt University Medical Center (Nashville, Tennessee), and SARS-CoV-2–positive specimens are sent to the University of Michigan (Ann Arbor, Michigan) for whole genome sequencing to identify SARS-CoV-2 lineages. Demographic and clinical data are collected through EHR review and patient or proxy interview. COVID-19 vaccination history is ascertained from state or jurisdictional registries, EHRs, and self-report.

### Data Analysis

The VISION and IVY networks conducted separate VE analyses. In both analyses, immunocompetent adults aged ≥18 years who 1) had a medical encounter at an ED/UC (VISION only) or 2) were hospitalized (VISION and IVY) at a participating facility with CLI were included. Case-patients were those who received a positive SARS-CoV-2 molecular test result, and control patients were those who received a negative SARS-CoV-2 test result.^¶¶^ Participants were excluded if they 1) received a COVID-19 vaccine dose <7 days before their eligible ED/UC encounter or hospitalization; 2) received an updated COVID-19 vaccine dose <2 months after receiving a previous COVID-19 vaccine dose (to align with current Advisory Committee on Immunization Practices recommendations); 3) received a bivalent COVID-19 vaccine dose after September 10, 2023; 4) received an updated COVID-19 vaccine dose before September 13, 2023; or 5) received >1 updated COVID-19 vaccine dose.*** Case-patients were also excluded if they had received a positive influenza or respiratory syncytial virus (RSV) molecular test result at the time of their CLI encounter.^†††^ Because of potential confounding caused by the association between COVID-19 and influenza vaccination behaviors, control patients who received positive or indeterminant influenza test results were excluded from the primary analysis^§§§^ (*4*). A sensitivity analysis including these control patients was also conducted.

Odds ratios (ORs) and 95% CIs were estimated using multivariable logistic regression comparing persons who received an updated COVID-19 vaccine dose with those who did not, irrespective of the number of previous original or bivalent COVID-19 vaccine doses received (if any), among case-patients and control patients. VE models were adjusted for age, sex, race and ethnicity, calendar time, and geographic region.^¶¶¶^ VE was calculated as (1 − adjusted OR) × 100%. In the VISION network, VE was estimated for adults aged ≥18 years and by age group (18–64 and ≥65 years). In the IVY network, statistical power was limited among younger adults because of lower vaccination coverage and fewer COVID-19–associated hospitalizations among persons aged 18–64 years; therefore, VE against hospitalization was estimated only for adults aged ≥18 years and ≥65 years.

Analyses were conducted using R software (version 4.3.2; R Foundation) for the VISION analysis and SAS software (version 9.4; SAS Institute) for the IVY analysis. This activity was reviewed by CDC, deemed not research, and was conducted consistent with applicable federal law and CDC policy.**** This activity was reviewed and approved as a research activity by one VISION site.

## Results

### Updated COVID-19 VE Against COVID-19–Associated ED/UC Encounters, VISION Network

Among adults aged ≥18 years in the VISION network, 128,825 ED/UC encounters met inclusion criteria, including 17,229 case-patients and 111,596 control patients (Table 1). A total of 1,297 (8%) case-patients and 13,378 (12%) control patients had received an updated COVID-19 vaccine dose. VE against COVID-19–associated ED/UC encounters was 51% (95% CI = 47%–54%) in the first 7–59 days after an updated dose (median interval since updated dose = 33 days) and 39% (95% CI = 33%–45%) in the 60–119 days after an updated dose (median interval since updated dose = 74 days) (Table 2). Among adults aged 18-64 years, VE against COVID-19–associated ED/UC encounters was 52% (95% CI = 45%–58%) in the first 7–59 days after an updated dose (median interval since updated dose = 31 days) and 45% (95% CI = 34%–55%) in the 60–119 days after an updated dose (median interval since updated dose = 73 days). Among adults aged ≥65 years, VE against COVID-19–associated ED/UC encounters was 49% (95% CI = 44%–54%) in the first 7–59 days after an updated dose (median interval since updated dose = 33 days) and 37% (95% CI = 29%–44%) in the 60–119 days after an updated dose (median interval since updated dose = 74 days).

**TABLE 1:** Characteristics of emergency department or urgent care encounters and hospitalizations among immunocompetent adults aged ≥18 years with COVID-19–like illness, by SARS-CoV-2 test result status and CDC vaccine effectiveness network — VISION and IVY networks, September 2023–January 2024

Characteristic	VE network, no. (column %)					
	VISION			IVY		
	Total no. of patients	COVID-19 case-patients	COVID-19 control patients	Total no. of patients	COVID-19 case-patients	COVID-19 control patients
**All ED/UC encounters**	**128,825**	**17,229**	**111,596**	**—**	**—**	**—**
**COVID-19 vaccination status**						
No updated dose*	**114,150 (89)**	15,932 (92)	98,218 (88)	—	—	—
Updated dose, ≥7 days earlier	**14,675 (11)**	1,297 (8)	13,378 (12)	—	—	—
Updated dose, 7–59 days earlier	**10,197 (8)**	825 (5)	9,372 (8)	—	—	—
Updated dose, 60–119 days earlier	**4,478 (3)**	472 (3)	4,006 (4)	—	—	—
**Median age, yrs (IQR)**	**52 (34–71)**	54 (35–72)	52 (33–70)	—	—	—
**Age group, yrs**						
18–64	**85,121 (66)**	10,959 (64)	74,162 (66)	—	—	—
≥65	**43,704 (34)**	6,270 (36)	37,434 (34)	—	—	—
**Female sex**	**78,702 (61)**	10,292 (60)	68,410 (61)	—	—	—
**Race and ethnicity**						
Black or African American, NH	**13,252 (10)**	1,425 (8)	11,827 (11)	—	—	—
White, NH	**81,818 (64)**	11,594 (67)	70,224 (63)	—	—	—
Hispanic or Latino, any race	**18,664 (14)**	2,316 (13)	16,348 (15)	—	—	—
Other, NH^†^	**12,782 (10)**	1,590 (9)	11,192 (10)	—	—	—
Unknown^§^	**2,309 (2)**	304 (2)	2,005 (2)	—	—	—
**HHS region^¶^**						
1	**0 (—)**	0 (—)	0 (—)	—	—	—
2	**0 (—)**	0 (—)	0 (—)	—	—	—
3	**0 (—)**	0 (—)	0 (—)	—	—	—
4	**0 (—)**	0 (—)	0 (—)	—	—	—
5	**45,232 (35)**	5,907 (34)	39,325 (35)	—	—	—
6	**0 (—)**	0 (—)	0 (—)	—	—	—
7	**0 (—)**	0 (—)	0 (—)	—	—	—
8	**37,173 (29)**	7,466 (43)	29,707 (27)	—	—	—
9	**37,346 (29)**	2,829 (16)	34,517 (31)	—	—	—
10	**9,074 (7)**	1,027 (6)	8,047 (7)	—	—	—
No. of chronic medical condition categories**						
0	**93,347 (72)**	13,540 (79)	79,807 (72)	—	—	—
1	**25,509 (20)**	2,542 (15)	22,967 (21)	—	—	—
2	**6,619 (5)**	814 (5)	5,805 (5)	—	—	—
3	**2,439 (2)**	230 (1)	2,209 (2)	—	—	—
4	**720 (1)**	84 (<1)	636 (1)	—	—	—
≥5	**191 (<1)**	19 (<1)	172 (<1)	—	—	—
**Month of COVID-19–associated ED/UC encounter**						
Sep 2023	**9,787 (8)**	1,222 (7)	8,565 (8)	—	—	—
Oct 2023	**29,836 (23)**	3,521 (20)	26,315 (24)	—	—	—
Nov 2023	**33,988 (26)**	4,487 (26)	29,501 (26)	—	—	—
Dec 2023	**42,403 (33)**	6,289 (37)	36,114 (32)	—	—	—
Jan 2024	**12,811 (10)**	1,710 (10)	11,101 (10)	—	—	—
**SARS-CoV-2 JN.1 lineage predominant period** ^††^	**24,923 (19)**	3,597 (21)	21,326 (19)	—	—	—
**All hospitalizations**	**37,503**	**4,589**	**32,914**	**4,117**	**1,194**	**2,923**
**COVID-19 vaccination status**						
No updated dose*	**32,909 (88)**	4,194 (91)	28,715 (87)	**3,670 (89)**	1,100 (92)	2,570 (88)
Updated dose, ≥7 days earlier	**4,594 (12)**	395 (9)	4,199 (13)	**447 (11)**	94 (8)	353 (12)
Updated dose, 7–59 days earlier	**3,326 (9)**	270 (6)	3,056 (9)	**283 (7)**	57 (5)	226 (8)
Updated dose, 60–119 days earlier	**1,268 (3)**	125 (3)	1,143 (3)	**164 (4)**	37 (3)	127 (4)
**Median age, yrs (IQR)**	**71 (59–81)**	77 (67–84)	71 (58–81)	**68 (55–78)**	73 (61–82)	66 (53–76)
**Age group, yrs**						
18–64	**12,975 (35)**	976 (21)	11,999 (36)	**1,765 (43)**	371 (31)	1,394 (48)
≥65	**24,528 (65)**	3,613 (79)	20,915 (64)	**2,352 (57)**	823 (69)	1,529 (52)
**Female sex**	**20,083 (54)**	2,365 (52)	17,718 (54)	**2,127 (52)**	623 (52)	1,504 (51)
**Race and ethnicity**						
Black or African American, NH	**3,979 (11)**	346 (8)	3,633 (11)	**929 (23)**	226 (19)	703 (24)
White, NH	**26,499 (71)**	3,479 (76)	23,020 (70)	**2,358 (57)**	752 (63)	1,606 (55)
Hispanic or Latino, any race	**3,510 (9)**	354 (8)	3,156 (10)	**540 (13)**	133 (11)	407 (14)
Other, NH^†^	**3,112 (8)**	373 (8)	2,739 (8)	**153 (4)**	40 (3)	113 (4)
Unknown^§^	**403 (1)**	37 (1)	366 (1)	**137 (3)**	43 (4)	94 (3)
**HHS region^¶^**						
1	**0 (—)**	0 (—)	0 (—)	**892 (22)**	330 (28)	562 (19)
2	**0 (—)**	0 (—)	0 (—)	**291 (7)**	62 (5)	229 (8)
3	**0 (—)**	0 (—)	0 (—)	**41 (1)**	14 (1)	27 (1)
4	**0 (—)**	0 (—)	0 (—)	**525 (13)**	125 (10)	400 (14)
5	**17,479 (47)**	2,154 (47)	15,325 (47)	**484 (12)**	160 (13)	324 (11)
6	**0 (—)**	0 (—)	0 (—)	**518 (13)**	129 (11)	389 (13)
7	**0 (—)**	0 (—)	0 (—)	**138 (3)**	36 (3)	102 (3)
8	**6,982 (19)**	1,061 (23)	5,921 (18)	**759 (18)**	194 (16)	565 (19)
9	**11,252 (30)**	1,211 (26)	10,041 (31)	**323 (8)**	101 (8)	222 (8)
10	**1,790 (5)**	163 (4)	1,627 (5)	**146 (4)**	43 (4)	103 (4)
No. of chronic medical condition categories**						
0	**5,113 (14)**	643 (14)	4,470 (14)	**389 (9)**	98 (8)	291 (10)
1	**6,316 (17)**	624 (14)	5,692 (17)	**807 (20)**	237 (20)	570 (20)
2	**7,234 (19)**	847 (18)	6,387 (19)	**1,171 (28)**	340 (28)	831 (28)
3	**9,230 (25)**	1,255 (27)	7,975 (24)	**975 (24)**	290 (24)	685 (23)
4	**6,545 (17)**	830 (18)	5,715 (17)	**521 (13)**	156 (13)	365 (12)
≥5	**3,065 (8)**	390 (8)	2,675 (8)	**254 (6)**	73 (6)	181 (6)
**Month of COVID-19–associated hospitalization**						
Sep 2023	**2,960 (8)**	270 (6)	2,690 (8)	**322 (8)**	126 (11)	196 (7)
Oct 2023	**9,789 (26)**	1,011 (22)	8,778 (27)	**1,081 (26)**	352 (29)	729 (25)
Nov 2023	**10,439 (28)**	1,283 (28)	9,156 (28)	**1,021 (25)**	300 (25)	721 (25)
Dec 2023	**11,791 (31)**	1,674 (36)	10,117 (31)	**949 (23)**	230 (19)	719 (25)
Jan 2024	**2,524 (7)**	351 (8)	2,173 (7)	**744 (18)**	186 (16)	558 (19)
**SARS-CoV-2 JN.1 lineage predominant period** ^††^	**5,486 (15)**	807 (18)	4,679 (14)	**901 (22)**	231 (19)	670 (23)

**Abbreviations:** ED = emergency department; HHS = U.S. Department of Health and Human Services; IVY = Investigating Respiratory Viruses in the Acutely Ill; NH = non-Hispanic; UC = urgent care; VE = vaccine effectiveness; VISION = Virtual SARS-CoV-2, Influenza, and Other respiratory viruses Network.

* The “no updated dose” group included all eligible persons who did not receive an updated 2023–2024 COVID-19 vaccine dose, regardless of number of previous (i.e., original monovalent and bivalent) doses (if any) received.

^†^ For VISION, “Other, NH” race includes persons reporting NH ethnicity and any of the following for race: American Indian or Alaska Native, Asian, Native Hawaiian or other Pacific Islander, other races not listed, and multiple races; because of small numbers, these categories were combined. For IVY, “Other, NH” race includes Asian, Native American or Alaska Native, and Native Hawaiian or other Pacific Islander; because of small numbers, these categories were combined.

^§^ For VISION, “Unknown” includes persons with missing race and ethnicity in their electronic health records. For IVY, “Unknown” includes patients who self-reported their race and ethnicity as “Other” and those for whom race and ethnicity were unknown.

^¶^ Regions are defined by HHS. States included in each region are available at https://www.hhs.gov/about/agencies/iea/regional-offices/index.html. VISION network sites included were located as follows. *Region 5:* HealthPartners (Minnesota and Wisconsin) and Regenstrief Institute (Indiana); *Region 8*: Intermountain Healthcare (Utah) and University of Colorado (Colorado); *Region 9*: Kaiser Permanente Northern California (California); and *Region 10*: Kaiser Permanente Northwest (Oregon and Washington). IVY network sites were located as follows: *Region 1*: Baystate Medical Center (Springfield, Massachusetts), Beth Israel Deaconess Medical Center (Boston, Massachusetts), and Yale University (New Haven, Connecticut); *Region 2*: Montefiore Medical Center (New York, New York); *Region 3*: Johns Hopkins Hospital (Baltimore, Maryland); *Region 4*: Emory University Medical Center (Atlanta, Georgia), University of Miami Medical Center (Miami, Florida), Vanderbilt University Medical Center (Nashville, Tennessee), and Wake Forest University Baptist Medical Center (Winston-Salem, North Carolina); *Region 5*: Cleveland Clinic (Cleveland, Ohio), Hennepin County Medical Center (Minneapolis, Minnesota), Henry Ford Health (Detroit, Michigan), The Ohio State University Wexner Medical Center (Columbus, Ohio), and University of Michigan Hospital (Ann Arbor, Michigan); *Region 6*: Baylor Scott & White Medical Center (Temple, Texas) and Baylor University Medical Center (Dallas, Texas); *Region 7*: Barnes-Jewish Hospital (St. Louis, Missouri) and University of Iowa Hospitals (Iowa City, Iowa); *Region 8*: Intermountain Medical Center (Murray, Utah), UCHealth University of Colorado Hospital (Aurora, Colorado), and University of Utah (Salt Lake City, Utah); *Region 9*: Stanford University Medical Center (Stanford, California), Ronald Reagan UCLA Medical Center (Los Angeles, California), and University of Arizona Medical Center (Tucson, Arizona); and *Region 10*: Oregon Health & Science University Hospital (Portland, Oregon) and University of Washington (Seattle, Washington).

** VISION underlying condition categories included pulmonary, cardiovascular, cerebrovascular, musculoskeletal, neurologic, hematologic, endocrine, renal, and gastrointestinal. IVY underlying condition categories included pulmonary, cardiovascular, neurologic, hematologic, endocrine, renal, gastrointestinal, and autoimmune.

^††^ The JN.1 predominant period was considered to have started December 24, 2023.

**TABLE 2:** Effectiveness of updated 2023–2024 (monovalent XBB.1.5) COVID-19 vaccination against laboratory-confirmed COVID-19–associated emergency department or urgent care encounters, by age group — VISION network, September 2023–January 2024

Age group, yrs/COVID-19 vaccination dosage pattern	No. (column %)		Median interval since last dose for vaccinated persons, days (IQR)	VE %* (95% CI)
	COVID-19 case-patients	COVID-19 control patients		
**≥18**				
No updated dose^†^ (Ref)	15,932 (92)	98,218 (88)	669 (403–792)	Ref
Received updated dose	1,297 (8)	13,378 (12)	44 (26–64)	47 (44–50)
7–59 days earlier	825 (5)	9,372 (8)	33 (20–46)	51 (47–54)
60–119 days earlier	472 (3)	4,006 (4)	74 (66–83)	39 (33–45)
**18–64**				
No updated dose^†^ (Ref)	10,582 (97)	69,423 (94)	697 (480–832)	Ref
Received updated dose	377 (3)	4,739 (6)	42 (24–62)	50 (44–55)
7–59 days earlier	259 (2)	3,457 (5)	31 (19–45)	52 (45–58)
60–119 days earlier	118 (1)	1,282 (2)	73 (66–83)	45 (34–55)
**≥65**				
No updated dose^†^ (Ref)	5,350 (85)	28,795 (77)	509 (362–733)	Ref
Received updated dose	920 (15)	8,639 (23)	46 (27–66)	45 (41–49)
7–59 days earlier	566 (9)	5,915 (16)	33 (21–46)	49 (44–54)
60–119 days earlier	354 (6)	2,724 (7)	74 (66–83)	37 (29–44)

**Abbreviations:** Ref = referent group; VE = vaccine effectiveness; VISION = Virtual SARS-CoV-2, Influenza, and Other respiratory viruses Network.

* VE was calculated as (1 − odds ratio) × 100% with odds ratios calculated using multivariable logistic regression. For VISION, the odds ratio was adjusted for age, sex, race and ethnicity, geographic region, and calendar time (days since January 1, 2021).

^†^ The “no updated dose” group included all eligible persons who did not receive an updated 2023–2024 COVID-19 vaccine dose, regardless of number of previous (i.e., original monovalent and bivalent) doses (if any) received.

### Updated COVID-19 VE Against COVID-19–Associated Hospitalization, VISION and IVY Networks

**VISION network.** Among adults aged ≥18 years in the VISION network, 37,503 hospitalizations met criteria for inclusion in analyses, including 4,589 case-patients and 32,914 control patients (Table 3). A total of 395 (9%) case-patients and 4,199 (13%) control patients had received an updated COVID-19 vaccine dose. VE against COVID-19–associated hospitalization was 53% (95% CI = 46%–59%) in the first 7–59 days after an updated dose (median interval since updated dose = 32 days) and 50% (95% CI = 40%–59%) in the 60–119 days after an updated dose (median interval since updated dose = 73 days). Among patients aged ≥65 years, VE against COVID-19–associated hospitalization was 54% (95% CI = 47%–60%) in the first 7–59 days after an updated dose (median interval since updated dose = 32 days) and 50% (95% CI = 39%–59%) in the 60–119 days after an updated dose (median interval since updated dose = 73 days).

**TABLE 3:** Effectiveness of updated 2023–2024 (monovalent XBB.1.5) COVID-19 vaccination against laboratory-confirmed COVID-19–associated hospitalization among adults aged ≥18 years — VISION and IVY networks, September 2023–January 2024

VE network/Age group, yrs/COVID-19 vaccination dosage pattern	No. (column %)		Median interval since last dose for vaccinated persons, days (IQR)	VE %* (95% CI)
	COVID-19 case-patients	COVID-19 control patients		
**VISION (4,589 case-patients and 32,914 control patients)**				
**≥18**				
No updated dose^†^ (Ref)	4,194 (91)	28,715 (87)	627 (383 to 765)	Ref
Received updated dose	395 (9)	4,199 (13)	42 (24 to 62)	52 (47 to 57)
7–59 days earlier	270 (6)	3,056 (9)	32 (19 to 45)	53 (46 to 59)
60–119 days earlier	125 (3)	1,143 (3)	73 (66 to 81)	50 (40 to 59)
**18–64**				
No updated dose^†^ (Ref)	938 (96)	11,342 (95)	685 (447 to 829)	Ref
Received updated dose	38 (4)	657 (5)	38 (22 to 58)	43 (20 to 59)
7–59 days earlier	28 (3)	503 (4)	30 (19 to 44)	42 (14 to 61)
60–119 days earlier	10 (1)	154 (1)	74 (67 to 81)	45 (–6 to 71)^§^
**≥65**				
No updated dose^†^ (Ref)	3,256 (90)	17,373 (83)	549 (370 to 745)	Ref
Received updated dose	357 (10)	3,542 (17)	43 (25 to 62)	53 (47 to 58)
7–59 days earlier	242 (7)	2,553 (12)	32 (19 to 46)	54 (47 to 60)
60–119 days earlier	115 (3)	989 (5)	73 (66 to 81)	50 (39 to 59)
**IVY (1,194 case-patients and 2,923 control patients)**				
**≥18**				
No updated dose^†^ (Ref)	1,100 (92)	2,570 (88)	645 (387 to 781)	Ref
Received updated dose	94 (8)	353 (12)	47 (25 to 71)	43 (27 to 56)
7–59 days earlier	—	—	—	—
60–119 days earlier	—	—	—	—
**≥65**				
No updated dose^†^ (Ref)	747 (91)	1,284 (84)	573 (375 to 752)	Ref
Received updated dose	76 (9)	245 (16)	48 (26 to 72)	48 (31 to 61)
7–59 days earlier	—	—	—	—
60–119 days earlier	—	—	—	—

**Abbreviations:** IVY = Investigating Respiratory Viruses in the Acutely Ill; Ref = referent group; VE = vaccine effectiveness; VISION = Virtual SARS-CoV-2, Influenza, and Other respiratory viruses Network.

* VE was calculated as (1 − odds ratio) × 100% with odds ratios calculated using multivariable logistic regression. For VISION, the odds ratio was adjusted for age, sex, race and ethnicity, geographic region, and calendar time (days since January 1, 2021). For IVY, the odds ratio was adjusted for age, sex, race and ethnicity, calendar time in biweekly intervals, and U.S. Department of Health and Human Services region.

^†^ The “no updated dose” group included all eligible persons who did not receive an updated 2023–2024 COVID-19 vaccine dose, regardless of number of previous (i.e., original monovalent and bivalent) doses (if any) received.

^§^ Some estimates are imprecise, which might be due to a relatively small number of persons in each level of vaccination or case status. This imprecision indicates that the actual VE could be substantially different from the point estimate shown, and estimates should therefore be interpreted with caution. Additional data accrual could increase precision and allow more precise interpretation.

**IVY network.** Among adults aged ≥18 years in the IVY network, 4,117 met criteria for inclusion in analyses, including 1,194 case-patients and 2,923 control patients. A total of 94 (8%) case-patients and 353 (12%) control patients had received an updated COVID-19 vaccine dose. VE of an updated dose against COVID-19–associated hospitalization was 43% (95% CI = 27%–56%, median interval since updated dose = 47 days) among adults aged ≥18 years and 48% (95% CI = 31%–61%, median interval since updated dose = 48 days) among adults aged ≥65 years.

Including control patients who received positive or indeterminant influenza test results added 1,819 control patients to the VISION hospitalization analysis and including control patients who received positive or indeterminant influenza test results or had missing influenza test results added 511 control patients to the IVY hospitalization analysis (Supplementary Table 1, https://stacks.cdc.gov/view/cdc/148434). VE estimates in supplementary analyses including control patients who received positive or indeterminant influenza test results did not differ meaningfully from those in the main analyses for ED/UC encounters (Supplementary Table 2, https://stacks.cdc.gov/view/cdc/148435) or hospitalization (Supplementary Table 3, https://stacks.cdc.gov/view/cdc/148436). 

Whole genome sequencing data were available for SARS-CoV-2–positive specimens collected in the IVY network during September 21–December 15, 2023. Among 952 sequenced specimens, 154 (16%) had XBB.1.5–like spike proteins, 550 (58%) had EG.5–like spike proteins with an F456L substitution compared with XBB.1.5, 189 (20%) had HK.3–like spike proteins with L455F and F456L substitutions compared with XBB.1.5, 57 (6%) had JN.1–like spike proteins with more than30 substitutions compared with XBB.1.5, and two (<1%) had other spike proteins.^††††^ Similarly, among 18,316 SARS-CoV-2–positive specimens collected during the same period and sequenced by CDC as part of national genomic surveillance,^§§§§^ 2,624 (14%) had XBB.1.5–like spike proteins, 9,649 (53%) had EG.5–like spike proteins, 3,700 (20%) had HK.3–like spike proteins, 2,302 (13%) had JN.1–like spike proteins, and 41 (<1%) had other spike proteins.

## Discussion

During September 2023–January 2024, in two multisite VE networks, updated 2023–2024 COVID-19 vaccination provided significant protection against COVID-19–associated ED/UC encounters and hospitalization among immunocompetent adults, compared with not receiving an updated vaccine. The comparison group included both unvaccinated persons and persons who had received original monovalent or bivalent doses only; thus, these results support current CDC recommendations for updated COVID-19 vaccination, including among persons who have previously received original monovalent or bivalent COVID-19 vaccines and those who have never been vaccinated, irrespective of previous infection history (*1*).

Updated COVID-19 vaccines contain the spike antigen from the SARS-CoV-2 Omicron XBB.1.5 virus, which was the predominant variant circulating in the United States during the first half of 2023. Many other XBB lineages cocirculated during fall 2023 that had amino acid substitutions associated with increased escape from neutralizing antibodies, such as EG.5 and HK.3 (*5*). The JN.1 lineage, a descendent of Omicron BA.2.86, was first detected in the United States in September 2023^¶¶¶¶^ and accounted for approximately 65% of circulating lineages by the 2-week period ending January 6, 2024.***** As noted, JN.1 contains more than 30 substitutions in the spike protein compared with XBB.1.5, some of which might be associated with immune escape (*5*). Although studies have found that updated COVID-19 vaccines elicit broadly cross-protective neutralizing antibodies, including against XBB lineages and JN.1 (*5*–*7*), the pace and frequency with which new SARS-CoV-2 lineages have displaced predecessors underscores the need for ongoing monitoring of COVID-19 VE and for periodic COVID-19 vaccine antigen updates. These analyses include periods when XBB lineages and JN.1 cocirculated to varying degrees in the United States, indicating that receipt of updated vaccines provided protection against COVID-19–associated ED/UC encounters and hospitalization due to the variants cocirculating during this period.

Despite different populations, methods, and outcomes, estimates of the effectiveness of updated COVID-19 vaccines were aligned across the VISION and IVY analyses. VE estimates were also similar to those recently published from another CDC VE platform, which measured VE against symptomatic SARS-CoV-2 infection (*8*), and to a United Kingdom report,^†††††^ which measured VE against hospitalization among patients aged ≥65 years. Earlier estimates of the effectiveness of updated COVID-19 vaccines against hospitalization in older adults from Denmark (*9*) and the Netherlands (*10*) were somewhat higher than those observed in this analysis; however, this is likely due to a shorter interval since updated dose receipt among patients included in the European studies or to differences in study methods. Whereas the maximum interval since receipt of an updated dose was 25 days in the Danish report and 2 months in the Dutch report, persons in the VISION and IVY analyses could have received an updated dose up to 4 months earlier.

In the VISION analysis, there was evidence of waning effectiveness of updated COVID-19 vaccines against ED/UC encounters; however, COVID-19–associated hospitalization rates during the analysis period were relatively low compared with previous years, limiting the evaluation of waning VE against hospitalization and precluding estimation of VE against critical illness. Analyses from VISION and IVY during 2022–2023 showed substantial waning of COVID-19 VE against ED/UC encounters and hospitalization, with VE not significantly different from zero in some strata by 6 months after vaccination, although VE was more sustained against critical illness (*2*,*3*) (defined as receipt of invasive mechanical ventilation, intensive care unit admission, or death), with protection lasting well over 1 year after the most recent dose. Continued monitoring of the effectiveness of updated COVID-19 vaccines for expected waning against hospitalization and to determine the durability of VE against critical illness is needed.

### Limitations

The findings in this report are subject to at least five limitations. First, although case-patients were required to meet a CLI definition and to receive a positive SARS-CoV-2 test result, they might have visited EDs or UCs or been hospitalized for reasons other than COVID-19, which could have lowered VE estimates. Second, misclassification of vaccination status was possible, because state registries, EHRs, medical claims data, and self-report might not identify all updated COVID-19 vaccine doses administered, which would likely result in underestimation of VE. Third, analyses did not account for previous SARS-CoV-2 infection, which might provide protection against future COVID-19. VE should therefore be interpreted as the incremental benefit of an updated dose in a population with high levels of infection-induced immunity, vaccine-induced immunity, or both. Fourth, although analyses were adjusted for relevant confounders, residual confounding from other factors, including behavioral modifications to prevent SARS-CoV-2 exposure and outpatient antiviral treatment for COVID-19, is possible. Finally, sample size limitations precluded estimation of lineage-specific VE and stratification of VE by interval since updated dose receipt in the IVY analysis.

### Implications for Public Health Practice

In this analysis of the effectiveness of updated COVID-19 vaccines, receipt of an updated COVID-19 vaccine dose provided protection against COVID-19–associated ED/UC encounters and hospitalization among immunocompetent adults. CDC will continue monitoring VE of updated COVID-19 vaccines. These results support CDC recommendations for updated 2023–2024 COVID-19 vaccination. All persons aged ≥6 months should receive updated 2023–2024 COVID-19 vaccine.

## Footnotes

* https://www.cdc.gov/vaccines/imz-managers/coverage/covidvaxview/interactive/adult-coverage-vaccination.html

† https://covid.cdc.gov/covid-data-tracker/#datatracker-home (Accessed February 13, 2024).

§ The VISION analysis included ED/UC encounters and hospitalizations during September 21, 2023–January 9, 2024. The IVY analysis included hospitalized patients admitted during September 21, 2023–January 31, 2024.

¶ Sites from the CDC-funded VISION network that contributed data for this analysis were HealthPartners (Minnesota and Wisconsin), Intermountain Healthcare (Utah), Kaiser Permanente Northern California (California), Kaiser Permanente Northwest (Oregon and Washington), Regenstrief Institute (Indiana), and University of Colorado (Colorado).

** COVID-19–like illness diagnoses were obtained from *International Classification of Diseases, Tenth Revision* (ICD-10) discharge codes. The specific codes used were COVID-19 pneumonia: J12.81 and J12.82; influenza pneumonia: J09.X1, J10.0, J10.00, J10.01, J10.08, J11.0, J11.00, and J11.08; other viral pneumonia: J12*; bacterial and other pneumonia: J13, J14, J15*, J16*, J17, and J18*; influenza disease: J09*, J10.1, J10.2, J10.8*, J11.1, J11.2, and J11.8*; acute respiratory distress syndrome: J80; chronic obstructive pulmonary disease with acute exacerbation: J44.1; asthma acute exacerbation: J45.21, J45.22, J45.31, J45.32, J45.41, J45.42, J45.51, J45.52, J45.901, and J45.902; respiratory failure: J96.0*, J96.2*, and R09.2; other acute lower respiratory tract infections: J20*, J21*, J22, J40, J44.0, J41*, J42, J43*, J47*, J85, J85.0, J85.1, J85.2, J85.3, and J86*; acute and chronic sinusitis: J01* and J32*; acute upper respiratory tract infections: J00*, J02*, J03*, J04*, J05*, and J06*; acute respiratory illness signs and symptoms: R04.2, R05, R05.1, R05.2, R05.4, R05.8, R05.9, R06.00, R06.02, R06.03, R06.1, R06.2, R06.8, R06.81, R06.82, R06.89, R07.1, R09.0*, R09.1, R09.2, R09.3, and R09.8*; acute febrile illness signs and symptoms: R50*, R50.81, and R68.83; acute nonrespiratory illness signs and symptoms: M79.10, M79.18, R10.0, R10.1*, R10.2, R10.3*, R10.81*, R10.84, R10.9, R11.0, R11.10, R11.11, R11.15, R11.2, R19.7, R21*, R40.0, R40.1,R41.82, R43*, R51.9, R53.1, R53.81, R53.83, R57.9, and R65*; respiratory failure, unspecified: J96.9*; febrile convulsions: R56.0; viral and respiratory diseases complicating pregnancy, childbirth, and puerperium: O98.5*, O98.8*, O98.9*, and O99.5*. All ICD-10 codes with * include all child codes under the specific parent code. One VISION site, representing 19% of case-patients from VISION analyses, did not include the following codes in its definition: J96.9*, O98.5*, O98.8*, O98.9*, O99.5*, and R56.0.

†† Sites from the CDC-funded IVY network that contributed data for this analysis were Barnes-Jewish Hospital (St. Louis, Missouri), Baylor Scott & White Medical Center (Temple, Texas), Baylor University Medical Center (Dallas, Texas), Baystate Medical Center (Springfield, Massachusetts), Beth Israel Deaconess Medical Center (Boston, Massachusetts), Cleveland Clinic (Cleveland, Ohio), Emory University Medical Center (Atlanta, Georgia), Hennepin County Medical Center (Minneapolis, Minnesota), Henry Ford Health (Detroit, Michigan), Intermountain Medical Center (Murray, Utah), Johns Hopkins Hospital (Baltimore, Maryland), Montefiore Medical Center (New York, New York), Oregon Health & Science University Hospital (Portland, Oregon), Ronald Reagan UCLA Medical Center (Los Angeles, California), Stanford University Medical Center (Stanford, California), The Ohio State University Wexner Medical Center (Columbus, Ohio), UCHealth University of Colorado Hospital (Aurora, Colorado), University of Arizona Medical Center (Tucson, Arizona), University of Iowa Hospitals (Iowa City, Iowa), University of Miami Medical Center (Miami, Florida), University of Michigan Hospital (Ann Arbor, Michigan), University of Utah (Salt Lake City, Utah), University of Washington (Seattle, Washington), Vanderbilt University Medical Center (Nashville, Tennessee), Wake Forest University Baptist Medical Center (Winston-Salem, North Carolina), and Yale University (New Haven, Connecticut).

§§ In the IVY analysis, CLI was defined as one or more of the following signs and symptoms: fever, cough, shortness of breath, new or worsening findings on chest imaging consistent with pneumonia, or hypoxemia defined as SpO2 <92% on room air or supplemental oxygen to maintain SpO2 ≥92%. For patients on chronic oxygen therapy, hypoxemia was defined as SpO2 below baseline or an escalation of supplemental oxygen to maintain a baseline SpO2.

¶¶ In the IVY analysis, patients were also classified as case-patients if they received a positive SARS-CoV-2 antigen test result.

*** In the IVY analysis, patients were also excluded if they experienced illness onset after hospital admission or withdrew.

††† One VISION site, representing 19% of case-patients from VISION analyses, did not provide RSV test results; therefore, RSV coinfections could not be excluded from this site.

§§§ In the IVY analysis, patients with missing influenza test results were also excluded.

¶¶¶ VISION regression models were adjusted for age, sex, race and ethnicity, calendar day, and geographic region with age and calendar day included as natural cubic splines. IVY regression models were adjusted for age, sex, race and ethnicity, calendar time in biweekly intervals, and U.S. Department of Health and Human Services region.

**** 45 C.F.R. part 46.102(l)(2), 21 C.F.R. part 56; 42 U.S.C. Sect. 241(d); 5 U.S.C. Sect. 552a; 44 U.S.C. Sect. 3501 et seq.

†††† Sequences were grouped by spike amino acid sequence similarity to SARS-CoV-2 lineages circulating during fall 2023. XBB.1.5–like, EG.5–like, and HK.3–like spike sequences are similar to the XBB.1.5 spike sequence used in updated 2023–2024 COVID-19 vaccines, with EG.5–like spikes having the additional F456L substitution, and HK.3–like spikes having additional L455F and F456L substitutions. JN.1 represents viruses in the JN.1 Pango lineage. “Other” represents non-XBB.1.5–derived, non-JN.1 viruses detected during September 21–December 15, 2023.

§§§§ CDC national SARS-CoV-2 genomic surveillance includes samples sequenced by CDC and national testing laboratories contracted by CDC.

¶¶¶¶
https://www.cdc.gov/respiratory-viruses/whats-new/SARS-CoV-2-variant-JN.1.html

*****
https://covid.cdc.gov/covid-data-tracker/#variant-proportions

†††††
https://assets.publishing.service.gov.uk/media/65b3c8a3c5aacc000da683d3/vaccine-surveillance-report-2024-week-4.pdf
